# Editorial: Plant growth defense trade-offs: investigating growth-resistance balance

**DOI:** 10.3389/fpls.2023.1327920

**Published:** 2023-11-07

**Authors:** Xuming Wang

**Affiliations:** State Key Laboratory for Managing Biotic and Chemical Threats to the Quality and Safety of Agroproducts, Institute of Virology and Biotechnology, Zhejiang Academy of Agricultural Sciences, Hangzhou, China

**Keywords:** growth challenges, plant health, plant pathogens, defense mechanisms, growth-resistance balance

Plants, those green guardians silently populating our ecosystems, have long been engaged in a delicate balancing act. At the core of this equilibrium lies the intriguing interaction between their growth and defense mechanisms. In this editorial, we will delve into the fascinating trade-offs between plant growth and defense, a dynamic interplay that impacts the health of our crops, the resilience of ecosystems, and ultimately, our own food security.

As stewards of the environment, plants face an array of adversaries. From harmful pathogens like fungi and viruses to relentless herbivores, plants must employ defense mechanisms to survive. These mechanisms include the production of chemical compounds, the activation of specific genes, and the use of physical barriers. However, the very systems that shield them have significant implications for their growth.

To grasp the complexity of this trade-off, we turn our attention to four relevant research papers that focus on this crucial balance.

## Unveiling the secrets of lesion-mimic mutants in rice

In the first paper (Zhao et al.), the researchers delve into the realm of lesion-mimic mutants (LMMs) in rice. LMMs are mutants that spontaneously produce necrotic spots, a defense response against pathogens. The study explores LMM8, a rice mutant that exhibits a striking lesion-mimic phenotype. This research reveals that while LMM8’s defense response is enhanced by light, it also comes at a significant cost to plant growth. The mutant is shorter and exhibits inferior agronomic traits compared to the wild type. These growth trade-offs are accompanied by a reduction in photosynthetic pigments, chloroplast damage, and increased production of reactive oxygen species. The findings of this study underscore the delicate balance that rice plants must strike between growth and defense, offering crucial insights into future strategies to improve rice yield and resistance.

## M451: a beacon of hope for wheat farmers

The second research (Kardava et al.) focus takes us to the realm of wheat farming, a critical component of global food security. The destructive *Fusarium* spp. poses a significant threat to wheat crops. The study introduces M451, a novel antifungal agent, as a potential solution. While conventional fungicides often harm plant growth, M451 proves to be a game-changer. It not only controls Fusarium spp. but also enhances wheat growth and nutritional quality. This research celebrates the possibility of effective, targeted, and eco-friendly fungicides that restore balance to plant growth and defense.

## The dance of viruses in passion fruit defense

Our third research (Zhang et al.) focus transports us to the world of passion fruit defense. These viruses, CfOLV1-CgOLV1 and CfOLV2, unexpectedly emerge as allies in the battle against the devastating *Cucumber mosaic virus* (CMV). These viruses reduce CMV pathogenicity while also influencing various growth parameters and nutritional quality. The intricate dance between these viruses and their host reveals the inherent complexity of trade-offs in plant defense mechanisms.

## A novel virus redefining plant-pathogen dynamics

Our final research (Guo et al.) feature sheds light on a novel virus in *Colletotrichum fructicola*. This virus, CfOLV2, is associated with hypovirulence, affecting both growth and pathogenicity. It alters the host fungus, leading to increased appressorium formation, melanin production, and modulation of cell wall integrity. This research exemplifies the multifaceted relationship between viruses and their fungal hosts, offering deeper insights into the trade-offs involved in plant defense mechanisms.

These four research papers collectively highlight the complex and multifaceted nature of plant growth-defense trade-offs. As plants strive to fend off pathogens and threats, they allocate resources and energy to their defense mechanisms, which, in turn, influence their growth and overall health. This trade-off is an inherent challenge that plants face on their path to survival.

However, these studies also bring hope and innovation. They demonstrate that there is a delicate equilibrium between plant growth and defense, and this balance can be harnessed to improve agricultural practices. From novel antifungal agents to the potential use of viruses as allies, these findings offer promising solutions for sustainable and resilient agriculture.

Future research in this field will continue to unravel the complexities of plant-pathogen interactions and provide tools to find the right balance between growth and defense. This equilibrium is not only vital for global food security but also for the preservation of our ecosystems and the well-being of our planet.

In conclusion, the intricate dance between plant growth and defense, as revealed in these research papers, showcases the remarkable resilience and adaptability of the plant kingdom. These studies serve as a reminder that nature’s wisdom, as we strive for sustainability and abundance, may hold the key to a flourishing future. Balancing growth and defense is a challenge that nature has mastered over millennia, and it’s a challenge that we, as stewards of the environment, must continue to explore and understand.

## Author contributions

XW: Writing – original draft.

